# Risk factors for non-sentinel lymph node metastasis in breast cancer: a meta-analysis

**DOI:** 10.3389/fonc.2026.1844072

**Published:** 2026-10-29

**Authors:** Lan Xu, Kaiyue Wang, Ziwei Chen, Chunxia Deng, Hong Zhang

**Affiliations:** 1 Department of General Surgery, Affiliated Renhe Hospital of China Three Gorges University, Yichang, China; 2 Medical College, China Three Gorges University, Yichang, China; 3 Nursing Department, Minda Hospital of Hubei Minzu University, Enshi, China; 4 Hubei Provincial Key Laboratory of Occurrence and Intervention of Rheumatic Diseases, Minda Hospital of Hubei Minzu University, Enshi, China; 5 Hubei Provincial Clinical Medical Research Center for Nephropathy, Minda Hospital of Hubei Minzu University, Enshi, China

**Keywords:** breast cancer, meta-analysis, metastasis, non-sentinel lymph node, risk factors

## Abstract

**Objective:**

To systematically evaluate risk factors associated with non-sentinel lymph node (NSLN) metastasis in patients with sentinel lymph node (SLN)-positive breast cancer.

**Methods:**

A comprehensive literature search was performed in PubMed, Embase, Web of Science, and the Cochrane Library from database inception to November 12, 2025. Studies reporting multivariable analyses of risk factors for NSLN metastasis were included. Study quality was independently assessed by two reviewers using the Newcastle–Ottawa Scale (NOS). Pooled odds ratios (ORs) and 95% confidence intervals (CIs) were estimated using a random-effects model. Heterogeneity, sensitivity analyses, and publication bias were also evaluated. All statistical analyses were conducted using Stata version 15.0.

**Results:**

A total of 37 studies involving 13,304 patients were included. The meta-analysis demonstrated that SLN macrometastasis (OR = 6.07, 95% CI: 2.21–16.7), abnormal preoperative AUS findings (OR = 3.51, 95% CI: 2.25–5.47), extranodal extension of SLN (OR = 2.67, 95% CI: 2.07 -3.44), lymphovascular invasion (OR = 2.68, 95% CI: 2.18–3.29), perineural invasion (OR = 2.37, 95% CI: 1.18–4.76), tumor diameter > 2 cm (OR = 2.06, 95% CI: 1.52–2.78), clinical T stage ≥ T2 (OR = 2.44, 95% CI: 2.02–2.95), histologic grade ≥ 3 (OR = 1.76, 95% CI: 1.44–2.16), number of positive SLNs (OR = 2.32, 95% CI: 1.53–3.53), SLN positivity ratio ≥ 50% (OR = 2.17, 95% CI: 1.74–2.70), multifocality (OR = 2.68, 95% CI: 1.98–3.63), HER2 positivity (OR = 2.22, 95% CI: 1.63–3.01), and a high Ki-67 index (OR = 2.20, 95% CI: 1.13–4.25) were associated with an increased risk of NSLN metastasis.

**Conclusion:**

SLN tumor burden, abnormal preoperative axillary ultrasound findings, tumor aggressiveness, and intrinsic biological characteristics (e.g., HER2 status and Ki-67 index) are key predictors of NSLN metastasis. Given the limitations in the quality and quantity of the included studies, further high-quality, large-scale prospective studies are warranted to validate these findings and provide more robust evidence for individualized axillary management in breast cancer.

**Systematic Review Registration:**

https://www.crd.york.ac.uk/PROSPERO/, identifier CRD420261301905.

## 1 Introduction

Breast cancer is the most commonly diagnosed malignancy among women worldwide. According to GLOBOCAN 2022, approximately 2.3 million new cases of breast cancer were diagnosed globally, with an age-standardized incidence rate (ASIR) of 46.8 per 100,000, ranking first among female malignancies (1). Axillary lymph node status is a key determinant of staging, treatment decision-making, and prognosis in breast cancer (2). Compared with traditional axillary lymph node dissection (ALND), sentinel lymph node biopsy (SLNB) reduces surgical morbidity while maintaining staging accuracy (3, 4). However, in patients with positive sentinel lymph nodes (SLNs), whether completion ALND is necessary remains controversial. Several studies (5, 6) have shown that not all SLN-positive patients harbor NSLN metastasis; approximately 40%–70% of patients have no NSLN involvement after ALND, suggesting that routine ALND may result in overtreatment.

Against this background, accurate identification of risk factors for NSLN metastasis is essential for optimizing axillary management and minimizing unnecessary treatment. Previous studies have explored a range of potential predictors, including tumor-related, pathological, and SLN-related characteristics; however, the findings have been inconsistent. For example, some studies (7, 8) have reported that tumor size and lymphovascular invasion are independent risk factors for NSLN metastasis, whereas others (9, 10) have failed to demonstrate statistical significance in multivariable analyses. Similarly, the predictive value of factors such as the number of positive SLNs and SLN macrometastasis varies considerably across studies (11). In this study, we conducted a meta-analysis to systematically evaluate the risk factors for NSLN metastasis in breast cancer, with the aim of providing evidence to facilitate risk stratification, optimize axillary decision-making, and reduce overtreatment.

## 2 Materials and methods

### 2.1 Study design and protocol registration

This systematic review and meta-analysis was conducted and reported in accordance with the Preferred Reporting Items for Systematic Reviews and Meta-Analyses (PRISMA 2020) Statement. The review protocol was registered in the International Prospective Register of Systematic Reviews (PROSPERO; Registration No. CRD420261301905) before data extraction and statistical analysis were initiated. The review objectives, eligibility criteria, outcomes, and analytical methods remained consistent with the registered protocol, and no substantial deviations occurred during the conduct of this review.

### 2.2 Search strategy

A systematic literature search was conducted in PubMed, Embase, Web of Science, and the Cochrane Library from database inception to November 12, 2025. Both Medical Subject Headings (MeSH) terms and free-text terms were used to improve the sensitivity and comprehensiveness of the search. The MeSH terms included “Breast Neoplasms,” “Lymphatic Metastasis,” and “Risk Factors,” combined with free-text terms such as “breast cancer,” “non-sentinel lymph node,” “lymph node metastasis,” “risk factor,” and “predictor.” The detailed search strategy for PubMed is provided in **Supplementary Table S1**. In addition, the reference lists of included studies were manually screened to identify potentially relevant articles.

### 2.3 Inclusion and exclusion criteria

Inclusion criteria

Studies were included if they met the following criteria:

1. Patients with pathologically confirmed breast cancer;
2. The outcome of interest was NSLN metastasis;
3. Multivariable analyses were performed, reporting ORs with 95% CIs, or sufficient data were provided to calculate these estimates;
4. Study design: cohort studies.

Exclusion criteria

Studies were excluded if they met any of the following criteria:

1. Special types of breast cancer (e.g., inflammatory or pregnancy-associated breast cancer);
2. NSLN metastasis was not clearly reported, or relevant data were incomplete or unavailable;
3. Only univariable analysis was performed without multivariable results;
4. Studies that only validated prediction models or nomograms without providing original data on risk factors;
5. Duplicate publications or overlapping populations (the most comprehensive dataset was retained);
6. Non-original studies, including reviews, meta-analyses, conference abstracts, and case reports.

### 2.4 Study selection and data extraction

All retrieved records were imported into EndNote X9 for management and duplicate removal. Two reviewers independently screened the studies according to the inclusion and exclusion criteria through title and abstract screening and full-text review. Discrepancies were resolved by discussion or consultation with a third reviewer. Data were extracted using a standardized form, including: first author, publication year, country, study design, sample size, number of NSLN-positive cases, patient characteristics (e.g., age and stage), regression models, adjustment strategies, and covariates. Detailed information on the regression model, adjustment strategy, and covariates retained in the final adjusted multivariable model is summarized in **Supplementary Table S2**. Only adjusted ORs and 95% CIs from multivariable analyses were extracted. If multiple models were reported in a single study, the model with the most comprehensive adjustment was selected.

### 2.5 Quality assessment

The quality of included studies was independently assessed by two reviewers using the Newcastle–Ottawa Scale (12). The NOS was selected because all included studies were observational cohort studies, for which it is a widely accepted tool to evaluate methodological quality. Disagreements were resolved through discussion or consultation with a third reviewer. The NOS evaluates studies across three domains: selection of study groups, comparability between groups, and outcome assessment, with a maximum score of 9 points. Studies scoring 7–9 were considered high quality, 4–6 moderate quality, and 0–3 low quality.

### 2.6 Statistical analysis

All statistical analyses were performed using Stata version 15.0. Odds ratios with 95% CIs were used as the effect measures to assess the association between potential risk factors and NSLN metastasis. Heterogeneity among studies was evaluated using the I² statistic and Cochran’s Q test, with I² values of 0%, 25%, 50%, and 75% representing no, low, moderate, and high heterogeneity, respectively.

Given potential variations in population characteristics, study design, and measurement methods across studies, a random-effects model was applied for all meta-analyses. Sensitivity analyses were conducted by sequentially excluding individual studies to assess the robustness of the results. Potential small-study effects were assessed using funnel plots and Egger’s test. When potential small-study effects were detected, the trim-and-fill method was applied to evaluate their potential impact on the pooled estimates. A two-sided *P* value < 0.05 was considered statistically significant.

## 3 Results

### 3.1 Literature search and study selection

A total of 406 records were initially identified through systematic database searching: PubMed (n = 71), Embase (n = 80), Cochrane Library (n = 26), and Web of Science (n = 229). After removing duplicates and records excluded by automated tools, 340 studies remained for title and abstract screening. Following the initial screening, 222 irrelevant studies were excluded, and 118 articles were assessed for full-text eligibility, of which four could not be retrieved. Ultimately, 114 full-text articles were evaluated, and 37 studies (13–49) were included in the systematic review and meta-analysis. The study selection process is illustrated in **Figure 1**.

**Figure 1:**
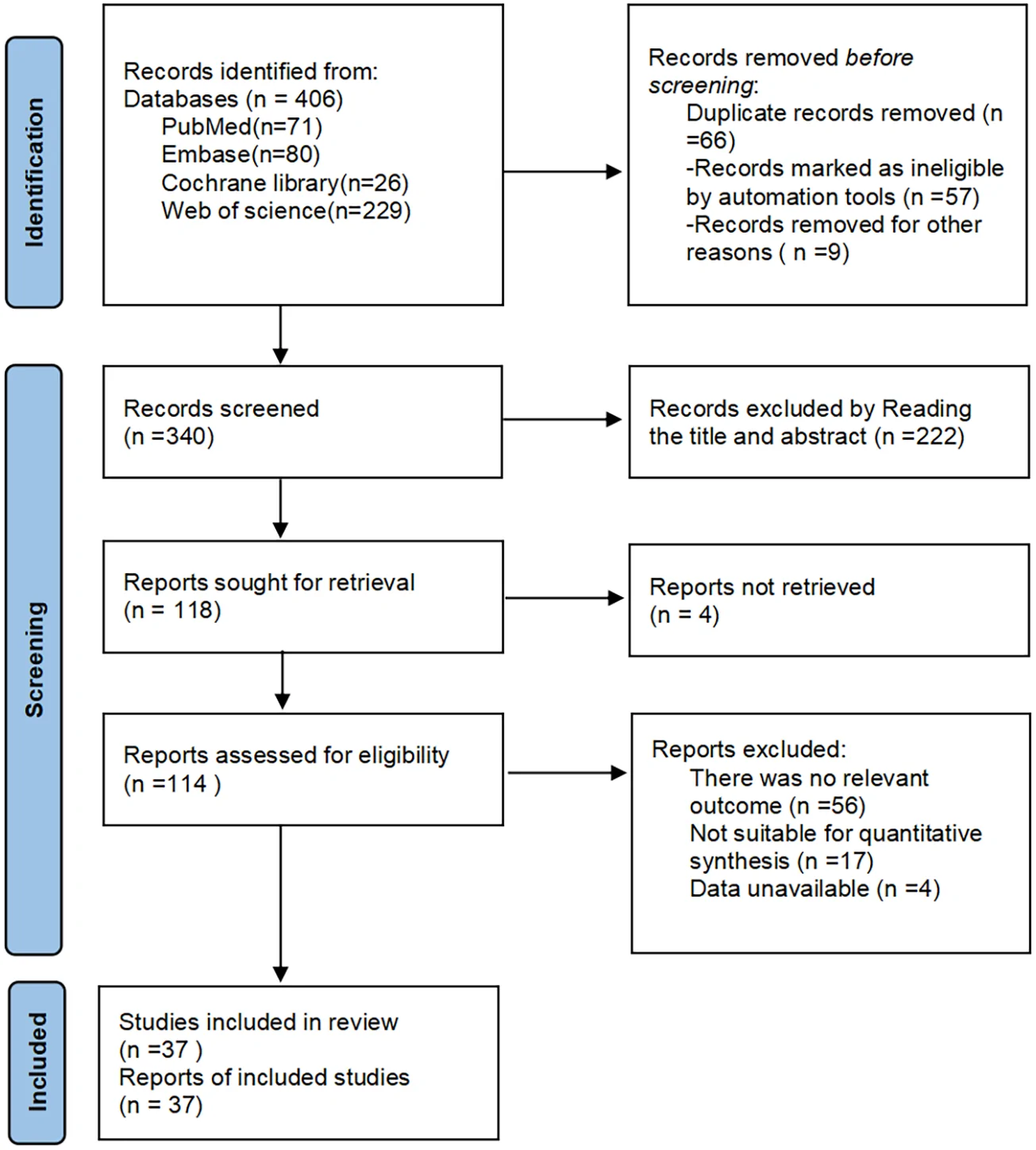
PRISMA flow diagram of the study selection process.

### 3.2 Characteristics and quality assessment

A total of 37 cohort studies involving 13,304 patients were included. The study populations were primarily from China, the United States, Canada, Turkey, Japan, and various European countries. The baseline characteristics of the included studies are summarized in **Table 1**.

**Table 1:** Baseline Characteristics.

Author	Year	Country	Study design	Total sample size	NSLN-positive patients	NAC exposure	Mean age (years)	Tumor stage	Regression model
Bolster	2007	The Netherlands	Prospective cohort study	186	56	NO	NR	T1–T2	Binary logistic regression
Cai SL	2022	China	Retrospective cohort study	443	167	NO	49.8 ± 10.8	T1–T2	Multivariable logistic regression
Chen J	2021	China	Retrospective cohort study	79	42	NO	54	cT1–T2	Multivariable logistic regression
Cserni G	2004	Hungary	Retrospective cohort study	150	60	NO	57.2	T1mic-T3	Multivariable logistic regression
Dave RV	2021	United Kingdom	Retrospective cohort study	2401	678	NO	NR	T1–T2	Multivariable logistic regression
Dong B	2018	China	Retrospective cohort study	509	194	NO	48.89	cT1-T2N0M0	Multivariable logistic regression
Dong L	2024	China	Retrospective cohort study	280	126	NO	50	NR	Multivariable logistic regression
Duan H	2023	China	Retrospective cohort study	118	82	NO	NR	NR	Multivariable logistic regression
Dyttert D	2016	Slovakia	Retrospective cohort study	274	90	NO	57.7±11.3	NR	Multivariable logistic regression
Fougo JL	2009	Portugal	Retrospective cohort study	143	66	NO	54	T1-T3	Multivariable logistic regression
Gurleyik G	2011	Turkey	Retrospective cohort study	23	13	NO	NR	T1-T3	Multivariable logistic regression
He Z	2019	China	Retrospective cohort study	206	117	NO	45	T1-T3	Multivariable logistic regression
Houvenaeghel G	2012	France	Multicenter retrospective cohort study	905	113	NO	NR	T0, T1, T2	Multivariable logistic regression
Huang J	2015	China	Retrospective cohort study	150	52	NO	51	NR	Multivariable logistic regression
Ishizuka Y	2020	Japan	Retrospective cohort study	184	64	NO	53.9 ± 12.6	T1-T3	Multivariable logistic regression
Kim GR	2018	South Korea	Retrospective cohort study	397	76	NO	NR	T1–T2	Multivariable logistic regression
Koca B	2014	Turkey	Retrospective cohort study	237	115	NO	49	T1-T3	Multivariable logistic regression
Kuo YL	2013	Taiwan, China	Retrospective cohort study	324	88	NO	NR	NR	Multivariable logistic regression
Kuru B	2016	Turkey	Retrospective cohort study	207	70	NO	49	T1–T2	Multivariable logistic regression
Kwon Y	2011	South Korea	Retrospective cohort study	205	89	NO	49.5	T1-T3	Multivariable logistic regression
Lale A	2020	Turkey	Retrospective cohort study	110	38	NO	52.64	cT1-2N0	Binary logistic regression
Liang M	2025	China	Retrospective cohort study	316	155	Yes	49	cT1-4N0-3	Multivariable logistic regression
Loza CM	2018	Argentina	Retrospective cohort study	348	182	NO	55.34	cT1–T2	Multivariable logistic regression
Moosavi SA	2014	Iran	Retrospective cohort study	167	92	NO	47.4 ±10.7	T1-T3	Multivariable logistic regression
Öz B	2018	Turkey	Retrospective cohort study	101	32	NO	52.4	T1-T3	Multivariable logistic regression
Ozmen V	2006	Turkey	Retrospective cohort study	148	75	NO	46	T1–T2	Multivariable logistic regression
Peyroteo M	2022	Portugal	Retrospective cohort study	328	161	NO	57.3	T1–T4	Multivariable logistic regression
Sanders SB	2022	United States	Retrospective cohort study	229	129	Yes	52	T1–T4	Multivariable logistic regression
Tapia G	2019	Australia	Retrospective cohort study	526	204	NO	56	T1-T3	Multivariable logistic regression
Wang XY	2019	China	Retrospective cohort study	396	90	NO	NR	T1–T2	Multivariable logistic regression
Wang Y	2023	China	Retrospective cohort study	83	42	NO	56.4	cT1-2N0M0	Multivariable logistic regression
Yang L	2024	China	Multicenter retrospective cohort study	830	229	NO	51.0 ± 11.0	cT1–T2	Multivariable logistic regression
Yang Y	2018	China	Retrospective cohort study	513	115	Yes	46.9±9.0	T1-T3	Multivariable logistic regression
Yu Y	2021	China	Multicenter retrospective cohort study	1150	345	NO	56.5±12.1	T1–T4	Multivariable logistic regression
Zhang W	2020	China	Retrospective cohort study	389	174	NO	51	cT1-2N0	Multivariable logistic regression
Zheng JW	2018	China	Retrospective cohort study	119	42	NO	52	T1–T2	Multivariable logistic regression
Zhou WB	2012	China	Retrospective cohort study	130	76	NO	50	T1-T3	Multivariable logistic regression

Age is presented as mean ± standard deviation unless otherwise specified; NR indicates not reported; Tumor stage was reported as clinical stage (cT) or pathological stage (pT) according to the original studies. NAC exposure: Yes indicates that all patients in the study received neoadjuvant chemotherapy before surgery; No indicates that no patients received neoadjuvant chemotherapy.

The quality of the included studies was independently assessed using the NOS. Among the included studies, 30 were rated as high quality (NOS score ≥7), and 7 were rated as moderate quality (NOS score 4–6). The detailed results of the quality assessment are presented in **Table 2**.

**Table 2:** Methodological quality of included studies based on the Newcastle-Ottawa Scale (NOS).

Study	Selection-4				Comparability-2		Outcome			Total scores
	Representativeness of the exposed cohort	Selection of the non exposed cohort	Ascertainment of exposure	Demonstration that outcome of interest was not present at start of study	Comparability of cohorts on the basis of the design or analysis		Assessment of outcome	Was follow-up long enough for outcomes to occur	Adequacy of follow up of cohorts	
					Select the most important factor	Study controls for any additional factor				
Bolster 2007 (13)	★	0	★	★	★	★	★	★	★	8
Cai SL 2022 (14)	★	0	★	★	★	0	★	★	★	7
Chen J 2021 (15)	★	0	★	★	★	0	★	★	0	6
Cserni G 2004 (16)	★	0	★	★	★	★	★	★	★	8
Dave RV 2021 (17)	★	0	★	★	★	★	★	★	★	8
Dong B 2018 (18)	★	0	★	★	★	★	★	★	★	8
Dong L 2024 (19)	★	0	★	★	★	0	★	★	★	7
Duan H 2023 (20)	★	0	★	★	★	★	★	★	★	8
Dyttert D 2016 (21)	★	0	★	★	★	★	★	★	★	8
Fougo JL 2009 (22)	★	0	★	★	★	0	★	★	★	7
Gurleyik G 2011 (23)	★	0	★	★	★	0	★	★	0	6
He Z 2019 (24)	★	0	★	★	★	★	★	★	★	8
Houvenaeghel G 2012 (25)	★	0	★	★	★	★	★	★	★	8
Huang J 2015 (26)	★	0	★	★	★	0	★	★	0	6
Ishizuka Y 2020 (27)	★	0	★	★	★	0	★	★	0	6
Kim GR 2018 (28)	★	0	★	★	★	★	★	★	0	7
Koca B 2014 (29)	★	0	★	★	★	★	★	★	0	7
Kuo YL 2013 (30)	★	0	★	★	★	★	★	★	★	8
Kuru B 2016 (31)	★	0	★	★	★	★	★	★	★	8
Kwon Y 2011 (32)	★	0	★	★	★	0	★	★	★	7
Lale A 2020 (33)	★	0	★	★	★	0	★	★	★	7
Liang M 2025 (34)	★	0	★	★	★	★	★	★	★	8
Loza CM 2018 (35)	★	0	★	★	★	★	★	★	★	8
Moosavi SA 2014 (36)	★	0	★	★	★	0	★	★	0	6
Öz B 2018 (37)	★	0	★	★	★	0	★	★	0	6
Ozmen V 2005	★	0	★	★	★	0	★	★	0	6
Peyroteo M 2022 (39)	★	0	★	★	★	★	★	★	★	8
Sanders SB 2022 (40)	★	0	★	★	★	★	★	★	★	8
Tapia G 2019 (41)	★	0	★	★	★	★	★	★	★	8
Wang XY 2019 (42)	★	0	★	★	★	★	★	★	★	8
Wang Y 2023 (43)	★	0	★	★	★	★	★	★	★	8
Yang L 2024 (44)	★	0	★	★	★	★	★	★	★	8
Yang Y 2018 (45)	★	0	★	★	★	★	★	★	0	7
Yu Y 2021 (46)	★	0	★	★	★	★	★	★	★	8
Zhang W 2020 (47)	★	0	★	★	★	★	★	★	★	8
Zheng JW 2018 (48)	★	0	★	★	★	★	★	★	★	8
Zhou WB 2012 (49)	★	0	★	★	★	★	★	★	★	8

### 3.3 Meta-analysis results

#### 3.3.1 Tumor diameter >2 cm

A total of 8 studies were included. Moderate heterogeneity was observed (I² = 56.1%, *P* = 0.025), and a random-effects model was applied. The pooled results showed that tumor diameter >2 cm was associated with an increased risk of NSLN metastasis (OR = 2.06, 95% CI: 1.52–2.78) (**Figure 2**).

**Figure 2:**
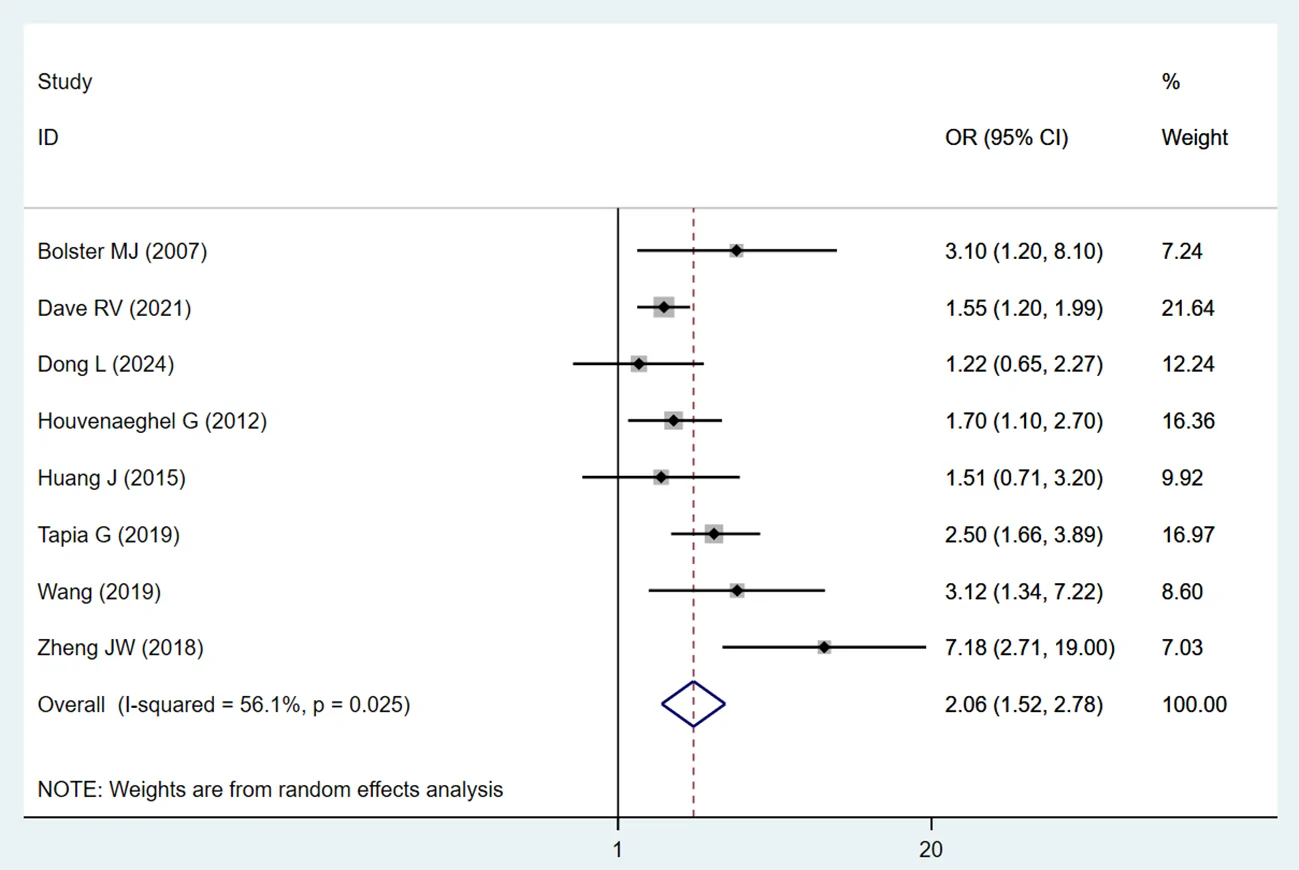
Forest plot for the association between tumor size > 2 cm and non-sentinel lymph node (NSLN) metastasis.

#### 3.3.2 Clinical T stage ≥T2

A total of 11 studies were included. No significant heterogeneity was observed (I² = 0.0%, *P* = 0.647), and a random-effects model was applied. The pooled results indicated that clinical T stage ≥T2 was associated with an increased risk of NSLN metastasis (OR = 2.44, 95% CI: 2.02–2.95) (**Figure 3**).

**Figure 3:**
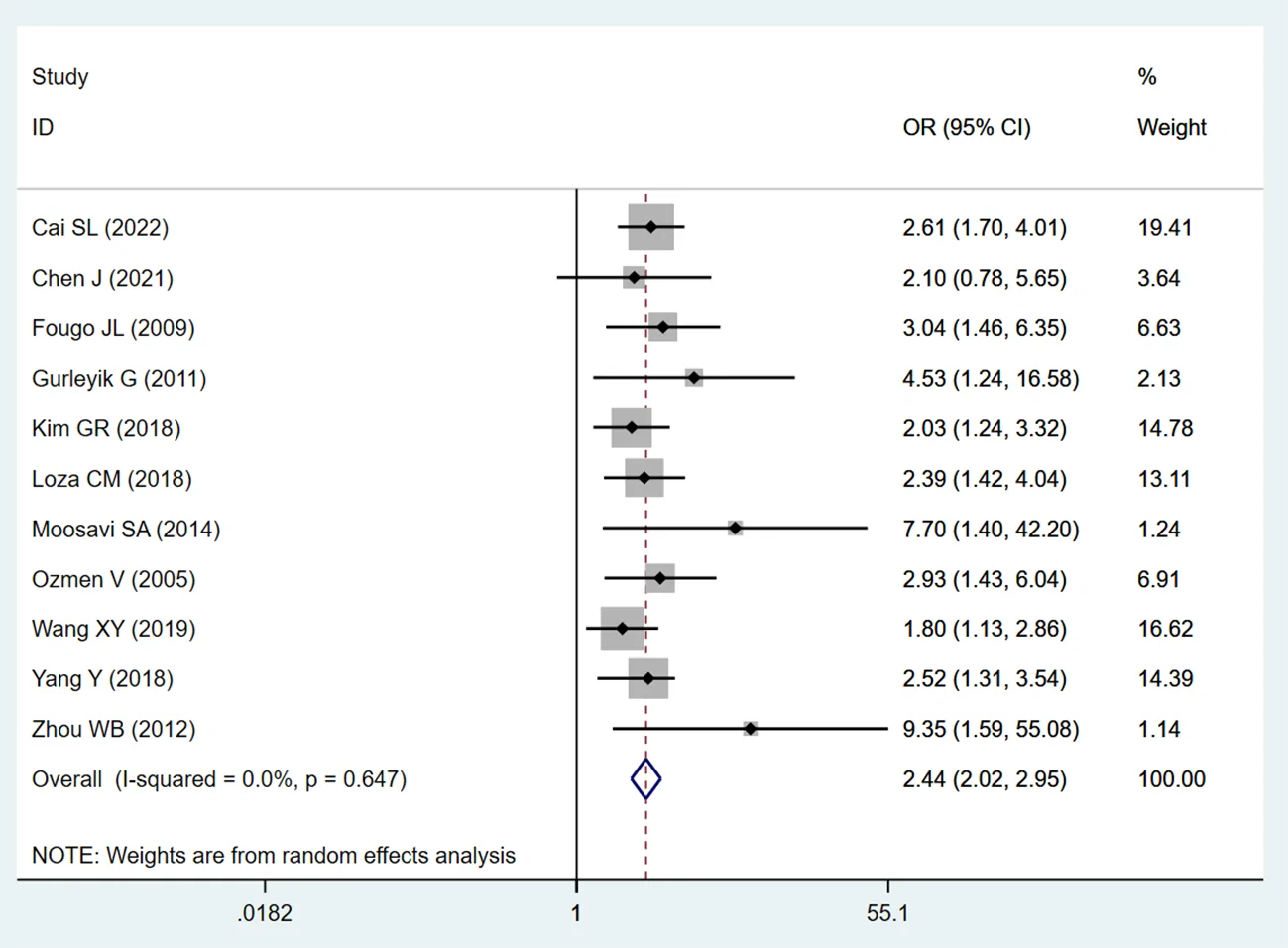
Forest plot for the association between clinical stage ≥ T2 and NSLN metastasis risk.

#### 3.3.3 Histologic grade ≥3

A total of 6 studies were included. No significant heterogeneity was observed (I² = 0.0%, *P* = 0.503), and a random-effects model was applied. The pooled results showed that histologic grade ≥3 was associated with an increased risk of NSLN metastasis (OR = 1.76, 95% CI: 1.44–2.16) (**Figure 4**).

**Figure 4:**
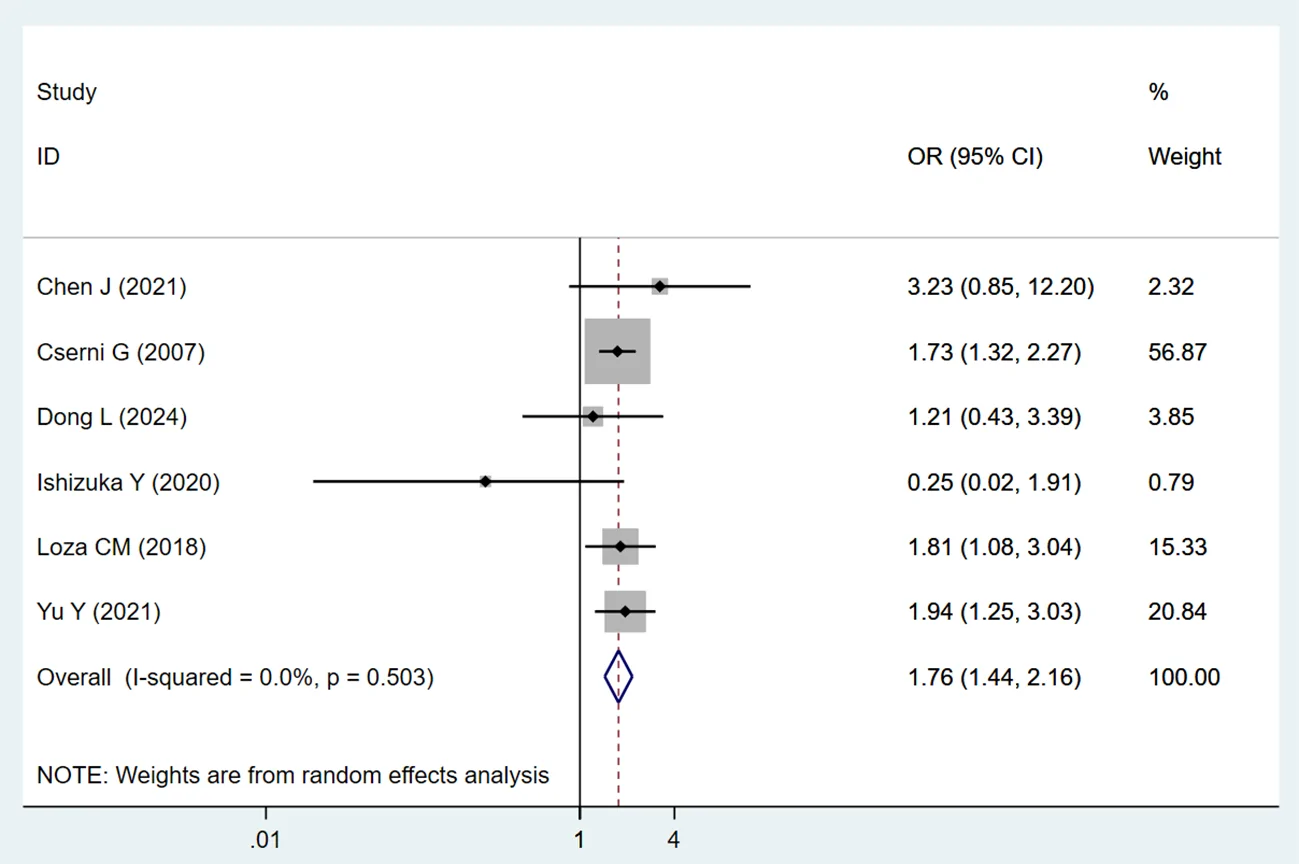
Forest plot for the association between histological grade ≥ 3 and NSLN metastasis.

#### 3.3.4 Extranodal extension

A total of 11 studies were included. Low heterogeneity was observed ( I² = 29.1%, P = 0.168), and a random-effects model was applied. The pooled results indicated that extranodal extension of SLN was associated with an increased risk of NSLN metastasis (OR = 2.67, 95% CI: 2.07 -3.44) (**Figure 5**).

**Figure 5:**
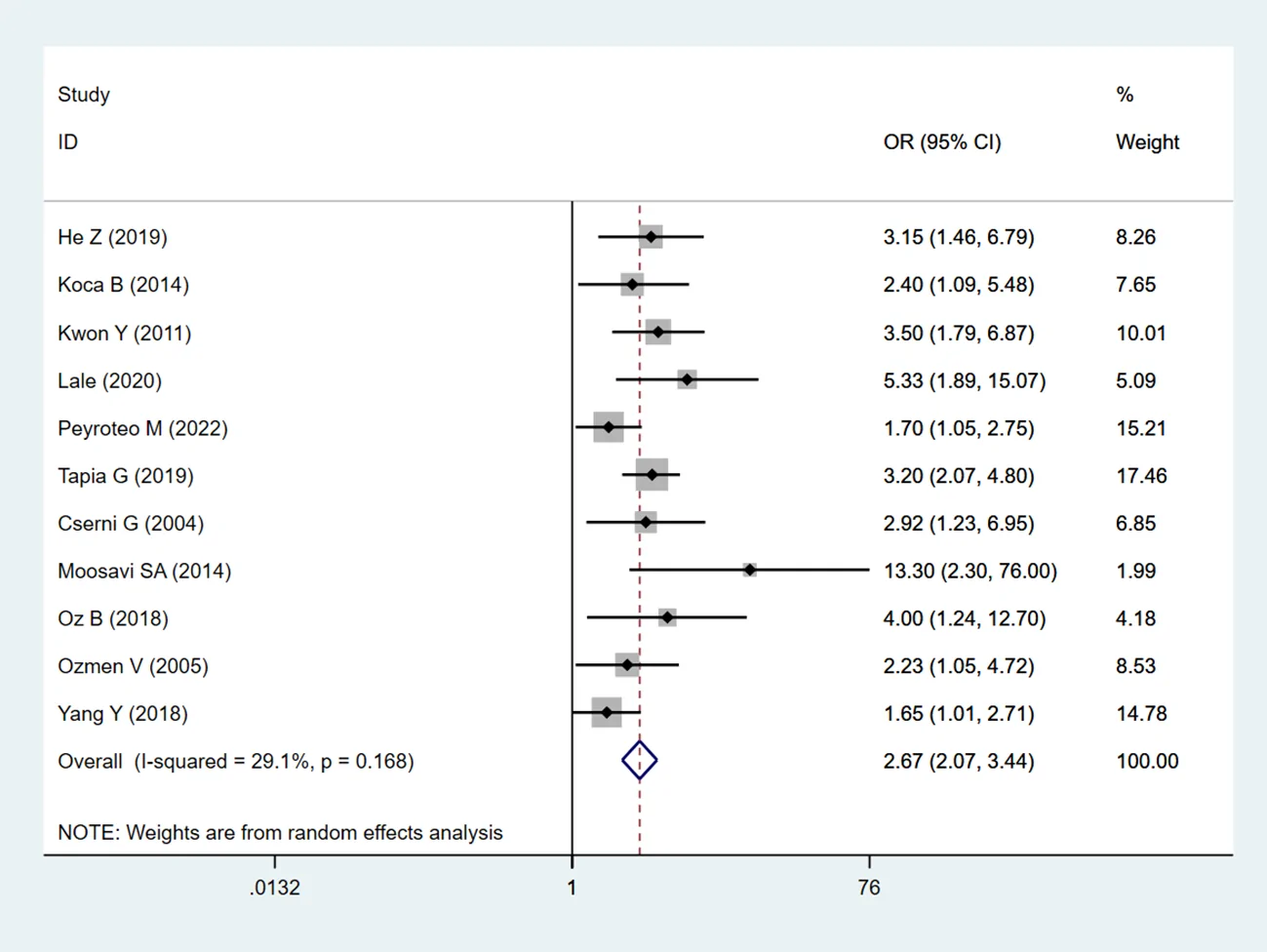
Forest plot for the association between sentinel lymph node extranodal extension and NSLN metastasis risk.

#### 3.3.5 Lymphovascular invasion

A total of 21 studies were included. Moderate heterogeneity was observed (I² = 48.2%, *P* = 0.007), and a random-effects model was applied. The pooled results showed that LVI positivity was associated with an increased risk of NSLN metastasis (OR = 2.68, 95% CI: 2.18–3.29) (**Figure 6**).

**Figure 6:**
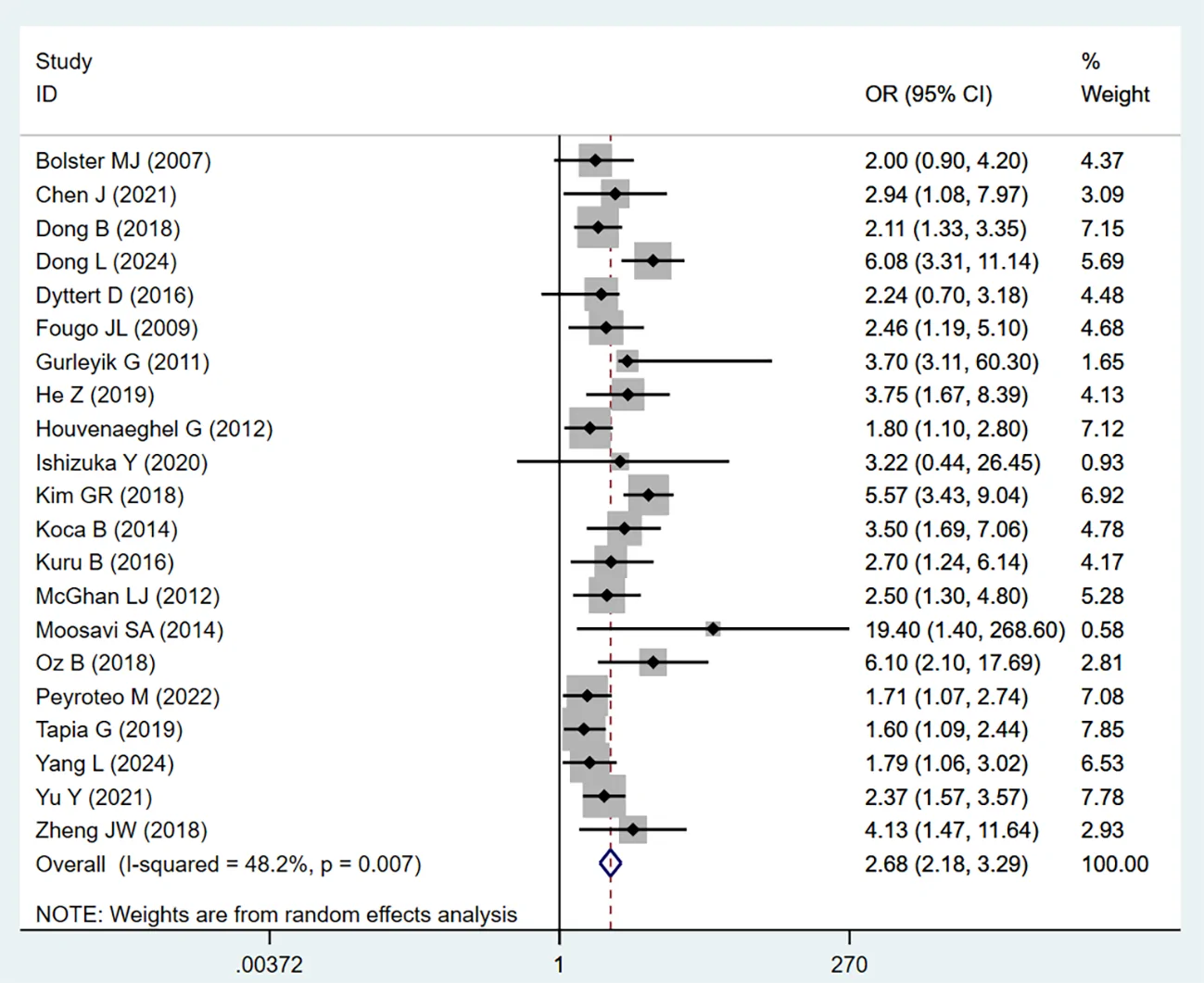
Forest plot of lymphovascular invasion (LVI) and NSLN metastasis risk.

#### 3.3.6 Perineural invasion

A total of 4 studies were included. Moderate heterogeneity was observed (I² = 54.7%, *P* = 0.085), and a random-effects model was applied. The pooled results indicated that PNI positivity was associated with an increased risk of NSLN metastasis (OR = 2.37, 95% CI: 1.18–4.76) (**Figure 7**).

**Figure 7:**
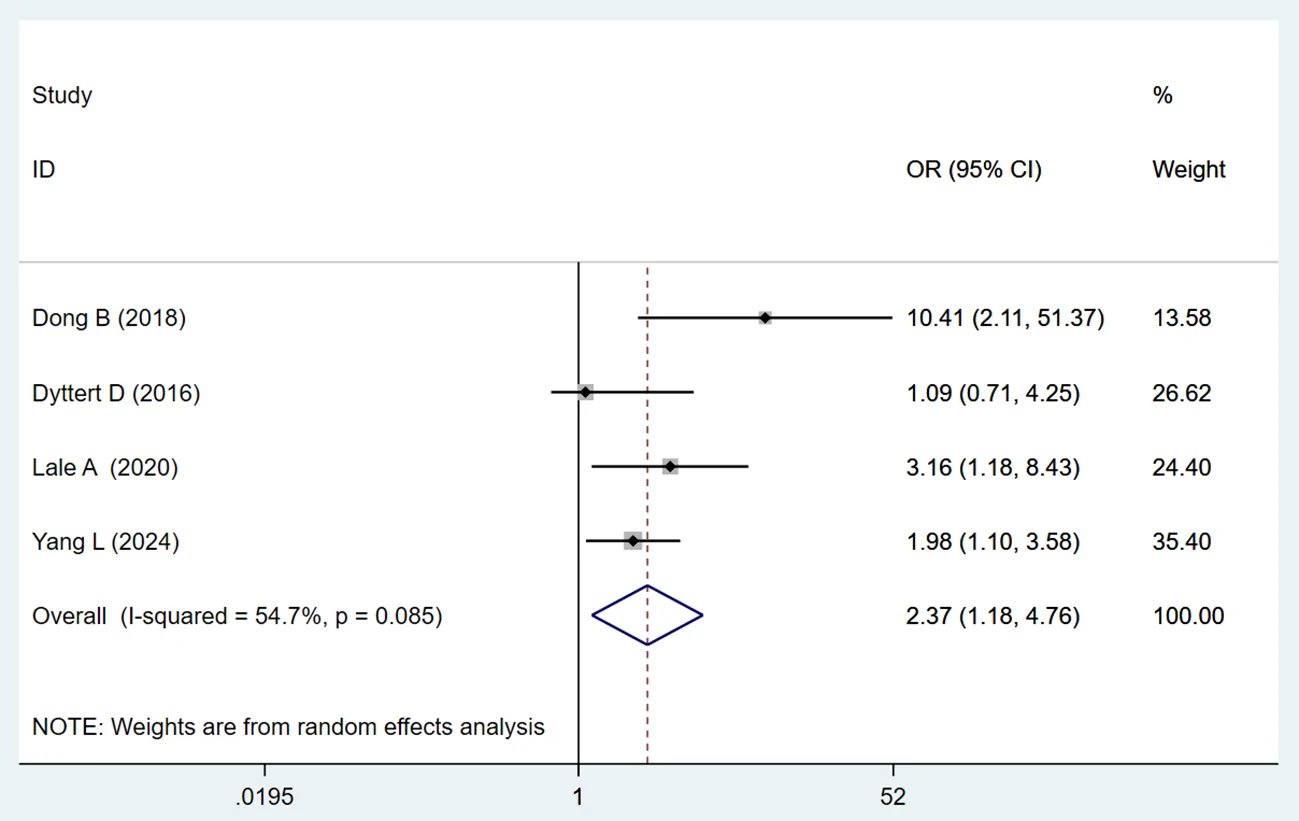
Forest plot of perineural invasion (PNI) and NSLN metastasis risk.

#### 3.3.7 SLN metastasis (macrometastasis vs micrometastasis)

A total of 5 studies were included. Moderate heterogeneity was observed (I² = 65.5%, *P* = 0.021), and a random-effects model was applied. The pooled results showed that, compared with micrometastasis, macrometastasis was associated with an increased risk of NSLN metastasis (OR = 6.07, 95% CI: 2.21–16.7) (**Supplementary Figure S2**).

#### 3.3.8 Number of positive SLNs (per additional node)

A total of 6 studies were included. Significant heterogeneity was observed (I² = 76.8%, *P =* 0.001), and a random-effects model was applied. The pooled results indicated that each additional positive SLN was associated with an increased risk of NSLN metastasis (OR = 2.32, 95% CI: 1.53–3.53) (**Supplementary Figure S3**).

#### 3.3.9 SLN positivity ratio (≥50% vs <50%)

A total of 9 studies were included. Low heterogeneity was observed (I² = 23.0%, P = 0.239), and a random-effects model was applied. The pooled results showed that an SLN positivity ratio ≥50% was associated with an increased risk of NSLN metastasis (OR = 2.17, 95% CI: 1.74–2.70) (**Supplementary Figure S4**).

#### 3.3.10 Multifocality

A total of 6 studies were included. No significant heterogeneity was observed (I² = 0.0%, *P =* 0.640), and a random-effects model was applied. The pooled results indicated that multifocality was associated with an increased risk of NSLN metastasis (OR = 2.68, 95% CI: 1.98–3.63) (**Supplementary Figure S5**).

#### 3.3.11 Estrogen receptor status

A total of 4 studies were included. Low to moderate heterogeneity was observed (I² = 42.6%, *P =* 0.156), and a random-effects model was applied. The pooled results showed that ER status was not significantly associated with NSLN metastasis (OR = 1.94, 95% CI: 0.95–3.95) (**Supplementary Figure S6**).

#### 3.3.12 Ki-67 index

A total of 4 studies were included. Moderate heterogeneity was observed (I² = 57.7%, *P =* 0.069), and a random-effects model was applied. The pooled results indicated that a high Ki-67 index (≥14%) was associated with an increased risk of NSLN metastasis (OR = 2.20, 95% CI: 1.13–4.25) (**Supplementary Figure S7**).

#### 3.3.13 Abnormal axillary lymph nodes on ultrasound (present vs absent)

A total of 5 studies were included. Moderate heterogeneity was observed (I² = 58.0%, *P =* 0.049), and a random-effects model was applied. The pooled results showed that abnormal axillary lymph nodes on preoperative ultrasound were associated with an increased risk of NSLN metastasis (OR = 3.51, 95% CI: 2.25–5.47) (**Supplementary Figure S8**).

#### 3.3.14 HER2 status

A total of 8 studies were included. No significant heterogeneity was observed (I² = 3.7%, *P* = 0.401), and a random-effects model was applied. The pooled results indicated that HER2-positive patients had a higher risk of NSLN metastasis than HER2-negative patients (OR = 2.22, 95% CI: 1.63–3.01) (**Supplementary Figure S9**).

### 3.4 Sensitivity analysis

Sensitivity analyses were performed for tumor diameter >2 cm, PNI, SLN metastasis, number of positive SLNs, Ki-67 index, and abnormal axillary ultrasound findings(**Supplementary Figures S10**–**S15**). The results showed that the direction and statistical significance of the pooled estimates remained unchanged after sequential exclusion of individual studies, indicating the robustness of the meta-analysis results.

### 3.5 Publication bias

Egger’s test and funnel plots (**Supplementary Figures S16**–**S20**) suggested potential small-study effects for clinical T stage ≥T2, LVI, SLN metastasis, number of positive SLNs, and HER2 status (*P* < 0.05). After adjustment using the trim-and-fill method (**Supplementary Figures S21**–**S25**), the pooled effect sizes were slightly attenuated; however, the direction and statistical significance of the results remained unchanged, indicating that the findings were robust and that these potential small-study effects had limited impact on the overall conclusions.

## 4 Discussion

This meta-analysis synthesized evidence from 37 studies involving 13,304 patients and identified multiple clinicopathological factors associated with NSLN metastasis in patients with positive sentinel lymph nodes. Indicators reflecting primary tumor burden, local invasiveness, axillary tumor burden, tumor biological characteristics, and preoperative imaging findings were all significantly associated with the risk of NSLN metastasis. Compared with previous evidence syntheses (50), the present study incorporated a larger and more contemporary evidence base and further evaluated emerging predictors, including HER2 status, Ki-67 index, and abnormal preoperative axillary ultrasound findings. These findings expand the current evidence regarding factors associated with NSLN metastasis; however, they should primarily be interpreted as evidence supporting risk stratification and hypothesis generation rather than direct guidance for clinical decision-making. In the contemporary era of axillary treatment de-escalation, the clinical value of these predictors lies in improving individualized estimation of nodal metastatic burden within carefully selected patient populations rather than determining the need for axillary lymph node dissection in isolation.

At the level of primary tumor burden, tumor diameter >2 cm, clinical T stage ≥T2, histologic grade ≥3, and multifocality were all associated with an increased risk of NSLN metastasis. These findings suggest that both the extent of the primary tumor and its intrinsic biological aggressiveness contribute to the likelihood of further axillary dissemination. Larger tumors have a greater probability of invading the surrounding lymphatic vasculature and generating metastatic deposits, whereas higher histologic grade reflects increased proliferative capacity, genomic instability, and invasive potential. These findings are consistent with previous studies by Min et al. (51) and Sopik et al. (52), which demonstrated that increasing primary tumor burden is closely associated with a higher risk of axillary lymph node involvement. In addition, unlike the earlier meta-analysis by van la Parra et al. (50), multifocality emerged as a significant predictor in the present study. This difference may reflect advances in breast imaging and pathological assessment over the past decade, leading to improved detection of multifocal disease and more accurate characterization of tumor distribution. Multifocal tumors may access multiple lymphatic drainage pathways simultaneously, thereby increasing the opportunity for tumor cells to disseminate beyond the sentinel lymph nodes to additional axillary nodes.

At the level of local invasiveness, LVI, ENE, and PNI were all associated with an increased risk of NSLN metastasis, indicating that disruption of anatomical barriers and enhanced invasive capability facilitate further regional lymphatic dissemination. LVI represents the direct invasion of tumor cells into lymphatic vessels and provides the initial pathway for metastatic spread. Early evidence from Weiser et al. (53) demonstrated that incorporating LVI substantially improved the prediction of NSLN metastasis. ENE reflects extension of metastatic tumor cells beyond the lymph node capsule, suggesting more aggressive tumor behavior and impaired containment by the host immune microenvironment. Multiple studies have consistently demonstrated that ENE is associated with both NSLN metastasis and a higher overall axillary nodal burden (54–56). Although fewer studies have evaluated PNI, increasing evidence suggests that neural invasion is associated with an aggressive tumor phenotype and may promote tumor dissemination through reciprocal interactions between tumor cells and the neural microenvironment (57).Collectively, these invasive pathological features reflect a greater capacity for regional spread and are likely to represent different manifestations of aggressive tumor biology rather than independent metastatic pathways.

At the level of axillary tumor burden, SLN macrometastasis, an increased number of positive SLNs, and a positivity ratio ≥50% were associated with an increased risk of NSLN metastasis. The extent of SLN involvement reflects the metastatic burden within the regional lymphatic system. The presence of macrometastasis or a greater number of positive SLNs indicates a higher lymphatic tumor burden and therefore a greater likelihood of metastatic spread beyond the sentinel nodes. Evidence from Zeng et al. (58) and Skarping et al. (59) further supports this interpretation, highlighting the value of quantitative assessment of SLN tumor burden in estimating the probability of additional axillary nodal metastasis and facilitating individualized risk stratification.

At the level of tumor biological characteristics and imaging features, HER2 positivity, a high Ki-67 index, and abnormal preoperative AUS findings were significantly associated with an increased risk of NSLN metastasis. HER2 overexpression has been shown to promote tumor cell proliferation, migration, and lymphatic dissemination through activation of multiple downstream signaling pathways, including the PI3K/AKT and MAPK pathways, while also stimulating lymphangiogenesis through upregulation of lymphangiogenic factors such as VEGF-C. Likewise, a high Ki-67 index reflects increased proliferative activity and aggressive tumor biology, which have consistently been associated with a greater propensity for regional lymph node metastasis. These findings support the biological plausibility of HER2 positivity and elevated Ki-67 as markers of increased metastatic potential. Abnormal preoperative AUS findings frequently indicate a higher likelihood of axillary nodal involvement and therefore provide valuable preoperative information for estimating nodal tumor burden. However, the diagnostic performance of AUS remains influenced by multiple factors (60, 61), including radiologist experience, imaging resolution, and treatment-related nodal changes following neoadjuvant chemotherapy (NAC), which may partially explain the variability observed across studies.

The clinical implications of the present findings should be interpreted within the context of the ongoing de-escalation of axillary management (62). Landmark randomized trials, including the ACOSOG Z0011, AMAROS, SENOMAC, and SINODAR-ONE trials, have demonstrated that omission of completion axillary lymph node dissection (ALND) in carefully selected patients with limited sentinel lymph node (SLN) involvement does not compromise locoregional recurrence, disease-free survival, or overall survival, thereby supporting less invasive approaches to axillary management (63–66). More recently, the SENOMAC trial further broadened the evidence base by enrolling a more heterogeneous patient population, including patients undergoing mastectomy, thereby improving the generalizability of axillary de-escalation strategies (65). Nevertheless, these trials were conducted in selected patient populations under predefined eligibility criteria, and caution is warranted when extrapolating their findings to patients encountered in routine clinical practice, particularly those presenting with a greater axillary tumor burden, receiving neoadjuvant chemotherapy, or otherwise falling outside current de-escalation criteria. In this context, accurate estimation of residual NSLN metastasis remains clinically relevant. Rather than being used in isolation to guide decisions regarding completion ALND, the clinicopathological predictors identified in the present meta-analysis provide evidence to support risk stratification through individualized estimation of residual axillary disease burden. These findings may also contribute to the development and validation of future prediction models, particularly for patients for whom the applicability of current de-escalation strategies remains uncertain. Further prospective studies are warranted to determine how these predictors can be incorporated into validated prediction models and evaluated for their potential role in clinical decision-making.

From a methodological perspective, interpretation of these findings should also consider the between-study heterogeneity observed for several predictors. Moderate to substantial heterogeneity was observed for SLN macrometastasis, the number of positive SLNs, Ki-67 index, and abnormal AUS findings. Notably, these predictors all depend on quantitative assessment, classification, or imaging interpretation and are therefore inherently more susceptible to methodological variation across studies. Among the predictors reflecting regional tumor burden, heterogeneity for SLN macrometastasis and the number of positive SLNs may partly reflect differences in pathological assessment, categorization of nodal tumor burden, patient selection criteria, and the inclusion of patients receiving NAC. Exploratory subgroup analyses according to NAC exposure yielded pooled estimates that were generally consistent with the primary analyses, suggesting that NAC alone is unlikely to explain the observed heterogeneity(**Supplementary Figures S1**–**S3**). Nevertheless, because only one or two studies contributed to each NAC subgroup, these findings should be interpreted cautiously. Methodological variability may also contribute to the heterogeneity observed for Ki-67 index and abnormal AUS findings. Although only studies using a 14% Ki-67 cutoff were quantitatively synthesized to improve comparability, many otherwise eligible studies adopted alternative thresholds (e.g., 20% or 30%) and therefore could not be included in the pooled analysis, limiting both the available evidence and further exploration of Ki-67-related heterogeneity. Likewise, the diagnostic performance of AUS is inherently influenced by imaging equipment, radiologist experience, interpretation criteria, and treatment-related nodal changes, all of which may contribute to variability across studies. Collectively, these findings suggest that the observed heterogeneity is unlikely to be attributable to a single factor but instead reflects the combined influence of clinical and methodological diversity across studies. Although most included studies adjusted for major clinicopathological confounders, differences in the covariates retained in the final multivariable models (**Supplementary Table S2**) may also have contributed to variation in the adjusted effect estimates. Because of the limited number of studies and inconsistent reporting of study-level characteristics, the relative contribution of each source of heterogeneity could not be quantified. Nevertheless, the consistency of the direction of effect across subgroup and sensitivity analyses strengthens confidence in the overall findings, suggesting that the observed heterogeneity is more likely to influence the precision of the pooled effect estimates rather than alter the overall direction of the identified associations.

### 5 Limitations

Several limitations should be acknowledged. First, the number of studies available for certain predictors was limited, which may have affected the stability of the pooled estimates. Second, most included studies were retrospective observational studies, introducing the possibility of selection bias, information bias, and residual confounding despite multivariable adjustment. Third, variability in variable definitions, cutoff values, and assessment methods across studies may have limited the comparability and generalizability of some pooled estimates. In addition, several pathological variables, including LVI, ENE, PNI, Ki-67, and histologic grade, are inherently subject to interobserver variability owing to differences in pathological interpretation, staining protocols, diagnostic criteria, and institutional practice, which may limit the reproducibility of these measurements across studies. Furthermore, although adjusted effect estimates were preferentially synthesized, differences in the covariates included in multivariable models across studies may still have influenced the pooled estimates. Assessment of small-study effects was not performed for predictors included in fewer than 10 studies because statistical tests for funnel plot asymmetry have limited power in such circumstances. Finally, although the Newcastle–Ottawa Scale (NOS) is widely used to assess the methodological quality of observational studies, it may not fully capture biases that are particularly relevant to prognostic factor research, including prognostic factor measurement, residual confounding, and statistical analysis. The pooled associations identified in this meta-analysis should not be interpreted as evidence of causality, and their clinical applicability should be further validated in large-scale, prospective, multicenter studies using standardized pathological and imaging assessment protocols. Future studies should also focus on integrating multidimensional clinicopathological predictors into externally validated prediction models to improve individualized risk stratification and facilitate their application across diverse patient populations.

## 6 Conclusion

This meta-analysis systematically evaluated the predictors of NSLN metastasis in breast cancer. The findings identified SLN macrometastasis, the number and ratio of positive SLNs, abnormal preoperative AUS findings, HER2 positivity, and a high Ki-67 index as independent predictors of NSLN metastasis. The risk of NSLN involvement appears to be driven by tumor anatomical burden, local invasive capacity, and intrinsic tumor biological characteristics. These findings provide a comprehensive synthesis of the current evidence and may support individualized risk stratification and estimation of residual axillary disease burden within a multidisciplinary treatment framework. However, the identified associations should not be interpreted as evidence of causality or used as stand-alone criteria for clinical decision-making. Further large-scale, prospective, multicenter studies are warranted to validate these findings and facilitate the development of robust prediction models for individualized axillary management.

## Data Availability

The original contributions presented in the study are included in the article/**Supplementary Material**. Further inquiries can be directed to the corresponding author/s.

## References

[1] Bray F Laversanne M Sung H Ferlay J Siegel RL Soerjomataram I . Global cancer statistics 2022: GLOBOCAN estimates of incidence and mortality worldwide for 36 cancers in 185 countries. CA: A Cancer J For Clin. (2024) 74:229–63. doi: 10.3322/caac.21834 38572751

[2] Chen MY Gillanders WE . Staging of the axilla in breast cancer and the evolving role of axillary ultrasound. Breast Cancer (Dove Med Press). (2021) 13:311–23. doi: 10.2147/bctt.s273039 34040436 PMC8139849

[3] Langer I Guller U Berclaz G Koechli OR Schaer G Fehr MK . Morbidity of sentinel lymph node biopsy (SLN) alone versus SLN and completion axillary lymph node dissection after breast cancer surgery: a prospective Swiss multicenter study on 659 patients. Ann Surg. (2007) 245:452–61. doi: 10.1097/01.sla.0000245472.47748.ec 17435553 PMC1877006

[4] Manca G Rubello D Tardelli E Giammarile F Mazzarri S Boni G . Sentinel lymph node biopsy in breast cancer: Indications, contraindications, and controversies. Clin Nucl Med. (2016) 41:126–33. doi: 10.1097/mnm.0000000000000489 26447368

[5] Padmanabhan N Ayub MF Hussain K Kurien A Radhakrishna S . Factors influencing non-sentinel node involvement in sentinel node positive patients and validation of MSKCC nomogram in Indian breast cancer population. Indian J Surg Oncol. (2015) 6:337–45. doi: 10.1007/s13193-015-0431-y 27065658 PMC4809847

[6] Gur AS Unal B Johnson R Ahrendt G Bonaventura M Gordon P . Predictive probability of four different breast cancer nomograms for nonsentinel axillary lymph node metastasis in positive sentinel node biopsy. J Am Coll Surg. (2009) 208:229–35. doi: 10.1016/j.jamcollsurg.2008.10.029 19228534

[7] Wei C Deng Y Wei S Huang Z Xie Y Xu J . Lymphovascular invasion is a significant risk factor for non-sentinel nodal metastasis in breast cancer patients with sentinel lymph node (SLN)-positive breast cancer: a cross-sectional study. World J Surg Oncol. (2023) 21:386. doi: 10.1186/s12957-023-03273-6 38097994 PMC10720167

[8] Huang Z Wu Z Zou QQ Xie YJ Li LH Huang YP . Risk factors of non-sentinel lymph node metastasis in breast cancer with 1-2 sentinel lymph node macrometastases underwent total mastectomy: a case-control study. World J Surg Oncol. (2023) 21:125. doi: 10.1186/s12957-023-02888-z 37024930 PMC10077622

[9] Mittendorf EA Hunt KK Boughey JC Bassett R Degnim AC Harrell R . Incorporation of sentinel lymph node metastasis size into a nomogram predicting nonsentinel lymph node involvement in breast cancer patients with a positive sentinel lymph node. Ann Surg. (2012) 255:109–15. doi: 10.1097/sla.0b013e318238f461 22167004 PMC4760742

[10] Maimaitiaili A Wu D Liu Z Liu H Muyiduli X Fan Z . Analysis of factors related to non-sentinel lymph node metastasis in 296 sentinel lymph node-positive Chinese breast cancer patients. Cancer Biol Med. (2018) 15:282–9. doi: 10.20892/j.issn.2095-3941.2018.0023 PMC6121045 30197795

[11] Ding J Jiang X Huang Z Ji Q Long J Cao Y . The prevalence of non-sentinel lymph node metastasis among breast cancer patients with sentinel lymph node involvement and its impact on clinical decision-making: a single-centred retrospective study. Oncol Rev. (2024) 18:1495133. doi: 10.3389/or.2024.1495133 39544961 PMC11560433

[12] Wells GA Shea B O'Connell D Peterson J Welch V Losos M . The Newcastle–Ottawa Scale (NOS) for assessing the quality of nonrandomised studies in meta-analyses. In: Ottawa Hospital Research Institute. Ottawa, Canada: Ottawa Hospital Research Institute (2011). Available online at: http://www.ohri.ca/programs/clinical_epidemiology/oxford.asp (Accessed March 20, 2026).

[13] Bolster MJ Peer PGM Bult P Thunnissen F Schapers RFM Meijer JWR . Risk factors for non-sentinel lymph node metastases in patients with breast cancer. The outcome of a multi-institutional study. Ann Surg Oncol. (2007) 14:181–9. doi: 10.1245/s10434-006-9065-1 17028772

[14] Cai SL Wei RM Han L Chen XG Gong GX Lin XQ . Risk factors of non-sentinel lymph node metastasis in 443 breast cancer patients with sentinel lymph node-positive. Medicine. (2022) 101:e29286. doi: 10.1097/md.0000000000029286 35866760 PMC9302317

[15] Chen J Luo X Cai G Li W Liu L Luo B . Study on the relationship between lymphovascular invasion and non-sentinel lymph node metastasis in early-stage breast cancer with positive sentinel lymph node. Cancer Res Clinic. (2021) 33:896–900. doi: 10.3760/cma.j.cn115355-20210410-00168

[16] Cserni G Burzykowski T Vinh-Hung V Kocsis L Boross G Sinkó M . Axillary sentinel node and tumour-related factors associated with non-sentinel node involvement in breast cancer. Jpn J Clin Oncol. (2004) 34:519–24. doi: 10.1093/jjco/hyh090 15466825

[17] Dave RV Cheung S Sibbering M Kearins O Jenkins J Gandhi A . Residual lymph node tumour burden following removal of a single axillary sentinel lymph with macrometastatic disease in women with screen-detected invasive breast cancer. BJS Open. (2021) 5(2):1–5. doi: 10.1093/bjsopen/zraa022 PMC7944503 33688940

[18] Dong BB Li Q Zhang MX Wu W Yao YD . Factors predicting non-sentinel lymph node status in breast cancer patients with 1-2 macrometastatic positive sentinel lymph nodes. Int J Clin Exp Med. (2018) 11:10836–46.

[19] Dong L Wei S Huang Z Liu F Xie Y Wei J . Association between postoperative pathological results and non-sentinel nodal metastasis in breast cancer patients with sentinel lymph node-positive breast cancer. World J Surg Oncol. (2024) 22:30. doi: 10.1186/s12957-024-03306-8 38268018 PMC10809690

[20] Duan HT Zhang JW Zhang GX Zhu XM Wang WJ . An improved nomogram including elastography for the prediction of non-sentinel lymph node metastasis in breast cancer patients with 1 or 2 sentinel lymph node metastases. Front Oncol. (2023) 13. doi: 10.3389/fonc.2023.1196592 37342193 PMC10277680

[21] Dyttert D Chvalny P Sabol M Waczulíková I Reke V Arciniegas L . Predictive factors of non-sentinel lymph node involvement in breast cancer patients with positive sentinel lymph nodes; a 5-year single centre experience. Eur Surgery-Acta Chirurgica Austriaca. (2016) 48:S125–9. doi: 10.1007/s10353-016-0400-x30311153

[22] Fougo JL Afonso M Senra FS Dias T Leal C Araújo C . Predictive factors for non-sentinel lymph node involvement in breast cancer patients with a positive sentinel node: Should we consider sentinel node-related factors? Clin Transl Oncol. (2009) 11:165–71. doi: 10.1007/s12094-009-0333-y 19293054

[23] Gurleyik G Aker F Aktekin A Saglam A . Tumor characteristics influencing non-sentinel lymph node involvement in clinically node negative patients with breast cancer. J Breast Cancer. (2011) 14:124–8. doi: 10.4048/jbc.2011.14.2.124 21847407 PMC3148535

[24] He Z Yu HY Yu HJ Wang YN Yang JM Li WP . Prediction model of non sentinel lymph node metastasis in sentinel lymph node positive breast cancer. Chin J Cancer Prev Treat. (2019) 26:1883–8. doi: 10.16073/j.cnki.cjcp.2019.24.12

[25] Houvenaeghel G Bannier M Nos C Giard S Mignotte H Jacquemier J . Non sentinel node involvement prediction for sentinel node micrometastases in breast cancer: Nomogram validation and comparison with other models. Breast. (2012) 21:204–9. doi: 10.1016/j.breast.2011.09.013 22014859

[26] Huang J Chen X Fei X Huang O Wu J Zong Y . Risk factors of non-sentinel lymph node metastasis and performance of MSKCC nomogramin breast cancer patients with metastatic sentinel lymph node. Zhonghua Wai Ke Za Zhi [Chinese J Surgery]. (2015) 53:941–6. doi: 10.3760/cma.j.issn.0529-5815.2015.12.011 26850667

[27] Ishizuka Y Horimoto Y Nakamura M Arakawa A Fujita T Iijima K . Predictive factors for non-sentinel nodal metastasis in patients with sentinel lymph node-positive breast cancer. Anticancer Res. (2020) 40:4405–12. doi: 10.21873/anticanres.14445 32727770

[28] Kim GR Choi JS Han BK Lee JE Nam SJ Ko EY . Preoperative axillary US in early-stage breast cancer: Potential to prevent unnecessary axillary lymph node dissection. Radiology. (2018) 288:55–63. doi: 10.1148/radiol.2018171987 29558291

[29] Koca B Kuru B Ozen N Yoruker S Bek Y . A breast cancer nomogram for prediction of non-sentinel node metastasis - Validation of fourteen existing models. Asian Pacific J Cancer Prev. (2014) 15:1481–8. doi: 10.7314/apjcp.2014.15.3.1481 24606487

[30] Kuo YL Chen WC Yao WJ Cheng LL Hsu HP Lai HW . Validation of Memorial Sloan-Kettering Cancer Center nomogram for prediction of non-sentinel lymph node metastasis in sentinel lymph node positive breast cancer patients an international comparison. Int J Surg. (2013) 11:538–43. doi: 10.1016/j.ijsu.2013.05.005 23707626

[31] Kuru B Sullu Y Yuruker S Bayrak IK Ozen N . Factors predicting non-sentinel lymph node metastasis in T1-2 invasive breast cancer with 1-2 axillary sentinel lymph node metastases: Presentation of Ondokuz Mayis scoring system. J Buon. (2016) 21:1129–36. 27837614

[32] Kwon Y Ro J Kang HS Kim SK Hong EK Khang SK . Clinicopathological parameters and biological markers predicting non-sentinel node metastasis in sentinel node-positive breast cancer patients. Oncol Rep. (2011) 25:1063–71. doi: 10.3892/or.2011.1157 21258771

[33] Lale A Yur M Özgül H Alkurt EG Yıldırım N Aygen E . Predictors of non-sentinel lymph node metastasis in clinical early stage (cT1-2N0) breast cancer patients with 1-2 metastatic sentinel lymph nodes. Asian J Surg. (2020) 43:538–49. doi: 10.1016/j.asjsur.2019.07.019 31519397

[34] Liang MT Zhu T Huang N Zhang LL Yang CQ Gao HF . Incorporating sentinel chain involvement pattern to predict non-sentinel lymph nodes status in breast cancer after neoadjuvant chemotherapy. Clin Transl Oncol. (2025) 28:203–14. doi: 10.1007/s12094-025-03993-z 40745244

[35] Loza CM Mandó P Ponce C Coló F Fabiano V Loza J . Predictive factors for non-sentinel lymph node metastasis in patients with ACOSOG Z0011 criteria. Breast Care. (2018) 13:434–8. doi: 10.1159/000488277 30800038 PMC6381814

[36] Moosavi SA Abdirad A Omranipour R Hadji M Razavi AE Najafi M . Clinicopathologic features predicting involvement of non-sentinel axillary lymph nodes in Iranian women with breast cancer. Asian Pacific J Cancer Prev. (2014) 15:7049–54. doi: 10.7314/apjcp.2014.15.17.7049 25227789

[37] Öz B Akcan A Dogan S Abdulrezzak Ü Aslan D Sözüer E . Prediction of nonsentinel lymph node metastasis in breast cancer patients with one or two positive sentinel lymph nodes. Asian J Surg. (2018) 41:12–9. doi: 10.1016/j.asjsur.2016.06.001 27591153

[38] Ozmen V Karanlik H Cabioglu N Igci A Kecer M Asoglu O . Factors predicting the sentinel and non-sentinel lymph node metastases in breast cancer. Breast Cancer Res Treat. (2006) 95:1–6. doi: 10.1007/s10549-005-9007-9 16322900

[39] Peyroteo M Canotilho R Correia AM Baía C Ribeiro C Reis P . Predictive factors of non-sentinel lymph node disease in breast cancer patients with positive sentinel lymph node. Cirugia Espanola. (2022) 100:81–7. doi: 10.1016/j.cireng.2022.01.003 35123939

[40] Sanders SB Hoskin TL Stafford AP Boughey JC . Factors influencing non-sentinel lymph node involvement in patients with positive sentinel lymph node(s) after neoadjuvant chemotherapy for breast cancer. Ann Surg Oncol. (2022) 29:7769–78. doi: 10.1245/s10434-022-12064-4 35834142

[41] Tapia G Ying V Di Re A Stellin A Cai TY Warrier S . Predicting non-sentinel lymph node metastasis in Australian breast cancer patients: Are the nomograms still useful in the post-Z0011 era? Anz J Surg. (2019) 89:712–7. doi: 10.1111/ans.15173 31066184

[42] Wang XY Wang JT Guo T Kong XY Chen L Zhai J . Risk factors and a predictive nomogram for non-sentinel lymph node metastases in Chinese breast cancer patients with one or two sentinel lymph node macrometastases and mastectomy. Curr Oncol (Toronto Ont). (2019) 26:e210–5. doi: 10.3747/co.26.4295 31043829 PMC6476451

[43] Wang Y Ling YW Jia LY Li KF Zhao J Zhao Y . A nomogram for intraoperatively predicting non-sentinel lymph node metastases in early breast cancer patients with positive sentinel lymph nodes. Gland Surg. (2023) 12:791–804. doi: 10.21037/gs-22-585 37441022 PMC10333765

[44] Yang L Zhao X Yang L Chang Y Cao C Li X . A new prediction nomogram of non-sentinel lymph node metastasis in cT1-2 breast cancer patients with positive sentinel lymph nodes. Sci Rep. (2024) 14:9596. doi: 10.1038/s41598-024-60198-0 38671007 PMC11053028

[45] Yang Y He YJ Fan ZQ Liu YQ Ouyang T . A new model to predict risk of nonsentinel lymph node status in Chinese sentinel lymph node-positive patients after neoadjuvant chemotherapy. Breast J. (2018) 24:883–8. doi: 10.1111/tbj.13065 29781094

[46] Yu Y Wang ZJ Wei ZY Yu BF Shen P Yan Y . Development and validation of nomograms for predicting axillary non-SLN metastases in breast cancer patients with 1-2 positive sentinel lymph node macro-metastases: a retrospective analysis of two independent cohorts. BMC Cancer. (2021) 21:466. doi: 10.21203/rs.3.rs-484084/v1 33902502 PMC8077841

[47] Zhang W Xu J Wang K Tang XJ Liang H He JJ . Independent risk factors for axillary lymph node metastasis in breast cancer patients with one or two positive sentinel lymph nodes. BMC Womens Health. (2020) 20:143. doi: 10.1186/s12905-020-01004-7 32646416 PMC7350751

[48] Zheng JW Cai SY Song HM Wang YL Han XF Wu HL . Positive non-sentinel axillary lymph nodes in breast cancer with 1-2 sentinel lymph node metastases. Medicine. (2018) 97:e13015. doi: 10.1097/md.0000000000013015 30383658 PMC6221619

[49] Zhou WB He ZY Xue JL Wang MH Zha XM Ling LJ . Molecular subtype classification is a determinant of non-sentinel lymph node metastasis in breast cancer patients with positive sentinel lymph nodes. PloS One. (2012) 7:e35881. doi: 10.1371/journal.pone.0035881 22563412 PMC3338552

[50] van la Parra RFD Peer PGM Ernst MF Bosscha K . Meta-analysis of predictive factors for non-sentinel lymph node metastases in breast cancer patients with a positive SLN. Ejso. (2011) 37:290–9. doi: 10.1016/j.ejso.2011.01.006 21316185

[51] Min SK Lee SK Woo J Jung SM Ryu JM Yu J . Relation between tumor size and lymph node metastasis according to subtypes of breast cancer. J Breast Cancer. (2021) 24:75–84. doi: 10.4048/jbc.2021.24.e4 33634622 PMC7920868

[52] Sopik V Narod SA . The relationship between tumour size, nodal status and distant metastases: on the origins of breast cancer. Breast Cancer Res Treat. (2018) 170:647–56. doi: 10.1007/s10549-018-4796-9 29693227 PMC6022519

[53] Weiser MR Montgomery LL Tan LK Susnik B Leung DY Borgen PI . Lymphovascular invasion enhances the prediction of non-sentinel node metastases in breast cancer patients with positive sentinel nodes. Ann Surg Oncol. (2001) 8:145–9. doi: 10.1245/aso.2001.8.2.145 11258779

[54] Nowikiewicz T Kurylcio A Głowacka-Mrotek I Szymankiewicz M Nowikiewicz M Zegarski W . Clinical relevance of a degree of extracapsular extension in a sentinel lymph node in breast cancer patients: a single-centre study. Sci Rep. (2021) 11:8982. doi: 10.1038/s41598-021-88351-z 33903665 PMC8076211

[55] Stitzenberg KB Meyer AA Stern SL Cance WG Calvo BF Klauber-DeMore N . Extracapsular extension of the sentinel lymph node metastasis: a predictor of nonsentinel node tumor burden. Ann Surg. (2003) 237:607–12. doi: 10.1097/01.sla.0000064361.12265.9a 12724626 PMC1514520

[56] Yang X Ma X Yang W Shui R . Clinical significance of extranodal extension in sentinel lymph node positive breast cancer. Sci Rep. (2020) 10:14684. doi: 10.1038/s41598-020-71594-7 32895434 PMC7477554

[57] Hosoya K Wakahara M Ikeda K Umekita Y . Perineural invasion predicts unfavorable prognosis in patients with invasive breast cancer. Cancer Diagnosis Prognosis. (2023) 3:208–14. doi: 10.21873/cdp.10203 36875309 PMC9949536

[58] Zeng X Li Y Sun C Liu Z Zhao J Ma X . A new model for predicting nonsentinel lymph node metastasis in early-stage breast cancer using MMP15. J Oncol. (2022) 2022:8675705. doi: 10.1155/2022/8675705 36035312 PMC9410952

[59] Skarping I Bendahl PO Szulkin R Alkner S Andersson Y Bergkvist L . Prediction of high nodal burden in patients with sentinel node-positive luminal ERBB2-negative breast cancer. JAMA Surg. (2024) 159:1393–403. doi: 10.1001/jamasurg.2024.3944 39320882 PMC11425194

[60] Schipper RJ van Roozendaal LM de Vries B Pijnappel RM Beets-Tan RG Lobbes MB . Axillary ultrasound for preoperative nodal staging in breast cancer patients: is it of added value? Breast. (2013) 22:1108–13. doi: 10.1016/j.breast.2013.09.002 24095611

[61] Dong Y Liu J Jia W Jia X Zhang J Zhu Y . Factors affecting preoperative sentinel lymph nodes contrast-enhanced ultrasound in breast cancer. Quant Imaging Med Surg. (2025) 15:8125–36. doi: 10.21037/qims-2024-2879 40893559 PMC12397643

[62] Fancellu A Giuliani G Mulas S Contini AM Ariu ML Sanna V . De-escalation of axillary treatment in early breast cancer—a narrative review of current trials. 2025. (2025) 6:5. doi: 10.21037/tbcr-24-45 39980812 PMC11836741

[63] Giuliano AE Ballman KV McCall L Beitsch PD Brennan MB Kelemen PR . Effect of axillary dissection vs no axillary dissection on 10-year overall survival among women with invasive breast cancer and sentinel node metastasis: the ACOSOG Z0011 (Alliance) randomized clinical trial. Jama. (2017) 318:918–26. doi: 10.1001/jama.2017.11470 28898379 PMC5672806

[64] Donker M van Tienhoven G Straver ME Meijnen P van de Velde CJH Mansel RE . Radiotherapy or surgery of the axilla after a positive sentinel node in breast cancer (EORTC 10981-22023 AMAROS): a randomised, multicentre, open-label, phase 3 non-inferiority trial. Lancet Oncol. (2014) 15:1303–10. doi: 10.1016/s1470-2045(14)70460-7 25439688 PMC4291166

[65] de Boniface J Filtenborg Tvedskov T Rydén L Szulkin R Reimer T Kühn T . Omitting axillary dissection in breast cancer with sentinel-node metastases. N Engl J Med. (2024) 390:1163–75. doi: 10.1056/nejmoa2313487 38598571

[66] Tinterri C Gentile D Gatzemeier W Sagona A Barbieri E Testori A . Preservation of axillary lymph nodes compared with complete dissection in T1-2 breast cancer patients presenting one or two metastatic sentinel lymph nodes: the SINODAR-ONE multicenter randomized clinical trial. Ann Surg Oncol. (2022) 29:5732–44. doi: 10.1245/s10434-022-11866-w 35552930

