# Supplementary material for: Risk factors for non-sentinel lymph node metastasis in breast cancer: a meta-analysis
Source: Front Oncol. 2026 Oct 29;16:1844072. doi: 10.3389/fonc.2026.1844072 (PMC13755699; doi:10.3389/fonc.2026.1844072)
Supplement: Supplementary file 1 [file Table1.docx]

Table S1. Search strategy for PubMed (searched on Nov 11, 2025)

**PubMed**

((((Breast Neoplasms[MeSH Terms]) OR ((((((((((((((((((((((((((((((((((((((Breast Neoplasms[Title/Abstract]) OR (Breast Neoplasm[Title/Abstract])) OR (Neoplasm, Breast[Title/Abstract])) OR (Neoplasms, Breast[Title/Abstract])) OR (Breast Tumors[Title/Abstract])) OR (Breast Tumor[Title/Abstract])) OR (Tumor, Breast[Title/Abstract])) OR (Tumors, Breast[Title/Abstract])) OR (Breast Cancer[Title/Abstract])) OR (Cancer, Breast[Title/Abstract])) OR (Cancer of Breast[Title/Abstract])) OR (Cancer of the Breast[Title/Abstract])) OR (Malignant Neoplasm of Breast[Title/Abstract])) OR (Breast Malignant Neoplasm[Title/Abstract])) OR (Breast Malignant Neoplasms[Title/Abstract])) OR (Malignant Tumor of Breast[Title/Abstract])) OR (Breast Malignant Tumor[Title/Abstract])) OR (Breast Malignant Tumors[Title/Abstract])) OR (Mammary Cancer[Title/Abstract])) OR (Cancer, Mammary[Title/Abstract])) OR (Cancers, Mammary[Title/Abstract])) OR (Mammary Cancers[Title/Abstract])) OR (Mammary Neoplasms, Human[Title/Abstract])) OR (Human Mammary Neoplasm[Title/Abstract])) OR (Human Mammary Neoplasms[Title/Abstract])) OR (Neoplasm, Human Mammary[Title/Abstract])) OR (Neoplasms, Human Mammary[Title/Abstract])) OR (Mammary Neoplasm, Human[Title/Abstract])) OR (Breast Carcinoma[Title/Abstract])) OR (Breast Carcinomas[Title/Abstract])) OR (Carcinoma, Breast[Title/Abstract])) OR (Carcinomas, Breast[Title/Abstract])) OR (Mammary Carcinoma, Human[Title/Abstract])) OR (Carcinoma, Human Mammary[Title/Abstract])) OR (Carcinomas, Human Mammary[Title/Abstract])) OR (Human Mammary Carcinomas[Title/Abstract])) OR (Mammary Carcinomas, Human[Title/Abstract])) OR (Human Mammary Carcinoma[Title/Abstract]))) AND ((Lymphatic Metastasis[MeSH Terms]) OR (((((Lymphatic Metastasis[Title/Abstract]) OR (Lymphatic Metastases[Title/Abstract])) OR (Lymph Node Metastasis[Title/Abstract])) OR (Lymph Node Metastases[Title/Abstract])) OR (Metastasis, Lymph Node[Title/Abstract])))) AND ((Risk Factors[MeSH Terms]) OR (((((((((((((((((((Risk Factors[Title/Abstract]) OR (Factor, Risk[Title/Abstract])) OR (Risk Factor[Title/Abstract])) OR (Population at Risk[Title/Abstract])) OR (Populations at Risk[Title/Abstract])) OR (Risk Scores[Title/Abstract])) OR (Risk Score[Title/Abstract])) OR (Score, Risk[Title/Abstract])) OR (Risk Factor Scores[Title/Abstract])) OR (Risk Factor Score[Title/Abstract])) OR (Score, Risk Factor[Title/Abstract])) OR (Health Correlates[Title/Abstract])) OR (Correlates, Health[Title/Abstract])) OR (Social Risk Factors[Title/Abstract])) OR (Factor, Social Risk[Title/Abstract])) OR (Factors, Social Risk[Title/Abstract])) OR (Risk Factor, Social[Title/Abstract])) OR (Risk Factors, Social[Title/Abstract])) OR (Social Risk Factor[Title/Abstract])))) AND ((((((non-sentinel[Title/Abstract]) OR (non sentinel[Title/Abstract])) OR (non sentinel lymph node[Title/Abstract])) OR (non-sentinel lymph node[Title/Abstract])) OR (non-sentinel nodal[Title/Abstract])) OR (non sentinel nodal[Title/Abstract]))

Supplementary Table S2. Regression models, adjustment strategies, and covariates retained in the final adjusted multivariable models of the included studies.

| **Study** | **Regression model** | **Adjustment strategy** | **Covariates included in the adjusted multivariable model** |
| --- | --- | --- | --- |
| **Bolster (2007)** | Multivariable logistic regression | **P<0.10 from univariable analysis** | **pN(sn); TS; LVI** |
| **Cai (2022)** | Stepwise multivariable logistic regression | **P<0.05 + stepwise** | **TS; SLNMT; pSLN; pSLNR** |
| **Chen（2021）** | Multivariable logistic regression | **NR** | **NR** |
| **Cserni (2004)** | Backward multivariable logistic regression | Backward elimination. | **NR** |
| **Dave (2021)** | Multivariable logistic regression | **Significant variables from univariable analysis** | **NR** |
| **Dong BB (2018)** | Multivariable logistic regression | NR | **Abnormal sonographic ALNs; Perineural invasion; cTNM stage; LVI; Ki67; pRatio** |
| **Dong L (2024)** | Multivariable logistic regression | NR | **NR** |
| **Duan (2023)** | Multivariable logistic regression | **Variables identified in univariable analysis** | **NR** |
| **Dyttert (2016)** | Prediction model based on multivariable logistic regression | **Significant + clinically relevant variables** | **NR** |
| **Fougo (2009)** | Multivariable logistic regression | **NR** | **Tumor size >20 mm; MF; LVI** |
| **Gurleyik (2011)** | Multivariable logistic regression | **NR** | **TS; LVI** |
| **He (2019)** | Multivariable logistic regression | **Significant variables from univariable analysis** | **NR** |
| **Houvenaeghel (2012)** | Multivariable logistic regression | **P<0.15 from univariable analysis** | TS; LVI; Detection method; **Mixed histology**; pSLNR |
| **Huang (2015)** | Multivariable logistic regression | **Significant variables from univariable analysis** | **NR** |
| **Ishizuka (2020)** | Multivariable logistic regression | **NR** | **NR** |
| **Kim GR (2018)** | Multivariable logistic regression | **NR** | **NR** |
| **Koca B (2014)** | Backward multivariable logistic regression | **Backward regression** | **pT; LVI; ENE; nSLN; SLNMS; MF** |
| **Kuo YL (2013)** | Backward multivariable logistic regression | **P<0.20 + backward** | **NR** |
| **Kuru B (2016)** | Backward multivariable logistic regression | **Significant variables + backward** | **pT; LVI; nSLN; SLNMS** |
| **Kwon Y (2011)** | **Multivariable logistic regression** | **NR** | **NR** |
| **Lale A (2020)** | **Binary multivariable logistic regression** | **P<0.05 from univariable analysis** | **HER2; PNI; SLNMS; ENE** |
| **Liang MT (2025)** | **Multivariable logistic regression** | **NR** | **SCIP; pSLN; nSLN; TS** |
| **Loza CM (2018)** | **Multivariable logistic regression** | **Significant variables from univariable analysis** | **HG; TS; Age** |
| **Moosavi SA (2014)** | **Multivariable logistic regression** | **Significant variables from univariable analysis** | **Age; TS; LVI; ENE; pSLNR** |
| **Öz B (2018)** | **Binary multivariable logistic regression** | **Significant variables from univariable analysis** | **LVI; ENE; HER2; Ki67** |
| **Ozmen V (2005)** | **Forward stepwise multivariable logistic regression** | **Forward stepwise** | **TS; SLNMS; ENE** |
| **Peyroteo M (2022)** | **Multivariable logistic regression** | **Significant variables from univariable analysis** | **LVI; ENE; SLNMS; pSLNR** |
| **Sanders SB, （2022）** | **Multivariable logistic regression** | **Best subset model selection** | **HER2; Multifocality; pSLN; SLNMS** |
| **Tapia G（2019）** | **Binary multivariable logistic regression** | **NR** | **ENE; TS; SLNMS; pSLNR; LVI** |
| **Wang XY（2019）** | **Backward stepwise binary logistic regression** | **Backward stepwise** | **TS; HG; nSLN; Imaging ALN** |
| **Wang Y（2023）** | **Multivariable logistic regression** | **Forward stepwise likelihood ratio** | **Age; pSLN; nSLN; TS; Monocyte count** |
| **Yang L（2024）** | **Multivariable logistic regression** | **NR** | **pSLN; pSLNR; LVI; PNI; ER** |
| **Yang Y（2018）** | **Backward stepwise binary logistic regression** | **Backward stepwise** | **Age; HR; Clinical response; TS; pSLN; SLNMS; ECE; Multifocality** |
| **Yu Y（2021）** | **Multivariable logistic regression** | **Best subset regression** | **Multiplicity; HG; TS; pSLN; nSLN; LVI** |
| **Zhang W（2020）** | **Multivariable logistic regression** | **Forward stepwise** | **Age; Occupation; pSLNR** |
| **Zheng JW（2018）** | **Multivariable logistic regression** | **Forward stepwise** | **LVI; TS; HER2; HG** |
| **Zhou WB（2012）** | **Multivariable logistic regression** | **NR** | **Age; TS; nSLN; SLNMS; HG; pSLNR; Molecular subtype** |

Abbreviations: Age, age; ALN, axillary lymph node; AUS, abnormal axillary ultrasonography finding; BMI, body mass index; CDFI, color Doppler flow imaging; ECE, extracapsular extension; ENE, extranodal extension; ER, estrogen receptor; HG, histological grade; HER2, human epidermal growth factor receptor 2; HR, hormone receptor; Ki67, Ki-67 proliferation index; LVI, lymphovascular invasion; MF, multifocality; Monocyte count, peripheral blood monocyte count; Molecular subtype, molecular subtype of breast cancer; nSLN, number of negative sentinel lymph nodes; pN(sn), pathological sentinel lymph node status; PNI, perineural invasion; pSLN, number of positive sentinel lymph nodes; pSLNR, ratio of positive to total sentinel lymph nodes; SCIP, sentinel chain involvement pattern; SLN, sentinel lymph node; SLNMS, sentinel lymph node metastasis size; SLNMT, sentinel lymph node metastasis type; TS, tumor size. NR, not reported. Covariates listed in the fourth column refer only to variables explicitly reported by the original study as being retained in the final adjusted multivariable model. When this information was unavailable, "NR" was recorded.

Table S3. Summary of robustness analyses for predictors with moderate to substantial heterogeneity

| **Predictor** | **No. of studies** | **Pooled OR**  **(95% CI)** | **I²**  **(%)** | **Small-study effects** | **Sensitivity analysis** | **Possible sources of heterogeneity** |
| --- | --- | --- | --- | --- | --- | --- |
| Tumor diameter >2 cm | 8 | 2.06 (1.52–2.78) | 56.1 | Not detected | Stable | Differences in patient characteristics and tumor size categorization |
| LVI | 21 | 2.68 (2.18–3.29) | 48.2 | Potential small-study effects; results unchanged after trim-and-fill | Stable | Variability in pathological assessment and multivariable adjustment strategies |
| PNI | 4 | 2.37 (1.18–4.76) | 54.7 | Not assessed | Stable | Limited number of studies and pathological assessment |
| SLN macrometastasis | 5 | 6.07 (2.21–16.70) | 65.5 | Potential small-study effects; results unchanged after trim-and-fill | Stable | Differences in pathological classification, patient selection, and NAC exposure |
| Number of positive SLNs | 6 | 2.32 (1.53–3.53) | 76.8 | Potential small-study effects; results unchanged after trim-and-fill | Stable | Variability in pathological assessment, nodal burden categorization, and NAC exposure |
| Ki-67 index | 4 | 2.20 (1.13–4.25) | 57.7 | Not assessed | Stable | Different cutoff values and assessment methods |
| Abnormal preoperative AUS | 5 | 3.51 (2.25–5.47) | 58 | Not assessed | Stable | Imaging equipment, radiologist experience, interpretation criteria, and treatment-related nodal changes |

**Abbreviations**: OR, odds ratio; CI, confidence interval; LVI, lymphovascular invasion; PNI, perineural invasion; SLN, sentinel lymph node; AUS, axillary ultrasound; NAC, neoadjuvant chemotherapy.

**Note:** Potential sources of heterogeneity were summarized based on the subgroup analyses, sensitivity analyses, and methodological considerations discussed in the manuscript. These potential sources should be interpreted as exploratory and hypothesis-generating rather than definitive explanations of heterogeneity. Small-study effect assessments based on fewer than 10 studies should be interpreted with caution because statistical tests for funnel plot asymmetry have limited power in such circumstances.

Figure S2. Forest plot of meta-analysis: Association between SLN metastasis status (macro- vs. micrometastasis) and NSLN metastasis risk.

Figure S3. Forest plot of the association between positive SLN number and NSLN metastasis.

Figure S4. Forest plot of the association between positive SLN ratio and NSLN metastasis.

Figure S5. Forest plot of the association between multifocality and NSLN metastasis.

Figure S6. Forest plot of the association between estrogen receptor status and NSLN metastasis.

Figure S7. Forest plot of the association between the Ki-67 index and NSLN metastasis.

Figure S8. Forest plot of the association between abnormal axillary lymph nodes on ultrasound and NSLN metastasis.

Figure S9. Forest plot of the association between HER2 status and NSLN metastasis.

Figure S10. Sensitivity analysis of tumor size > 2 cm.

Figure S11. Sensitivity analysis of perineural invasion (PNI).

Figure S12. Sensitivity analysis of the impact of SLN metastasis (macrometastasis vs. micrometastasis) .

Figure S13. Sensitivity analysis for the number of positive SLNs

Figure S14. Sensitivity analysis of the Ki-67 index

Figure S15. Sensitivity analysis of abnormal axillary lymph nodes on ultrasound

Figure S16. Funnel plot for clinical tumor stage ≥T2 and NSLN metastasis.

Figure S17. Funnel plot for lymphovascular invasion and NSLN metastasis.

Figure S18. Funnel plot for SLN metastasis burden and NSLN metastasis.

Figure S19. Funnel plot for the number of positive SLNs and NSLN metastasis.

Figure S20. Funnel plot for HER2 status and NSLN metastasis.

Figure S21.Trim-and-fill analysis for tumor clinical stage ≥T2 and NSLN metastasis.

Figure S22. Trim-and-fill analysis for lymphovascular invasion and NSLN metastasis.

Figure S23. Trim-and-fill analysis for SLN metastasis burden and NSLN metastasis.

Figure S24. Trim-and-fill analysis for positive SLN number and NSLN metastasis.

Figure S25. Trim-and-fill analysis for HER2 status and NSLN metastasis.

Figure S26. Subgroup analysis of the association between the number of positive sentinel lymph nodes and NSLN metastasis according to NAC exposure.

Figure S27. Subgroup analysis of the association between HER2 status and NSLN metastasis according to NAC exposure.

Figure S28. Subgroup analysis of the association between extranodal extension (ENE) and NSLN metastasis according to NAC exposure.
